# Ethical and Legal Challenges of Telemedicine in the Era of the COVID-19 Pandemic

**DOI:** 10.3390/medicina57121314

**Published:** 2021-11-30

**Authors:** Renata Solimini, Francesco Paolo Busardò, Filippo Gibelli, Ascanio Sirignano, Giovanna Ricci

**Affiliations:** 1 National Centre on Addiction and Doping, Istituto Superiore di Sanità, 00161 Rome, Italy; renata.solimini@iss.it; 2 Department of Excellence of Biomedical Science and Public Health, University “Politecnica delle Marche” of Ancona, 60126 Ancona, Italy; 3 School of Law, Camerino University, 62032 Camerino, Italy; filippo.gibelli@studenti.univr.it (F.G.); ascanio.sirignano@unicam.it (A.S.); giovanna.ricci@unicam.it (G.R.)

**Keywords:** telemedicine, telehealth, ethics, legislation, COVID-19

## Abstract

*Background and objective*: Telemedicine or telehealth services has been increasingly practiced in the recent years. During the COVID-19 pandemic, telemedicine turned into and indispensable service in order to avoid contagion between healthcare professionals and patients, involving a growing number of medical disciplines. Nevertheless, at present, several ethical and legal issues related to the practice of these services still remain unsolved and need adequate regulation. This narrative review will give a synthesis of the main ethical and legal issues of telemedicine practice during the COVID-19 pandemic. *Material and Methods*: A literature search was performed on PubMed using MeSH terms: Telemedicine (which includes Mobile Health or Health, Mobile, mHealth, Telehealth, and eHealth), Ethics, Legislation/Jurisprudence, and COVID-19. These terms were combined into a search string to better identify relevant articles published in the English language from March 2019 to September 2021. *Results*: Overall, 24 out of the initial 85 articles were considered eligible for this review. Legal and ethical issues concerned important aspects such as: informed consent (information about the risks and benefits of remote therapy) and autonomy (87%), patient privacy (78%) and confidentiality (57%), data protection and security (74%), malpractice and professional liability/integrity (70%), equity of access (30%), quality of care (30%), the professional–patient relationship (22%), and the principle of beneficence or being disposed to act for the benefit of others (13%). *Conclusions*: The ethical and legal issues related to the practice of telehealth or telemedicine services still need standard and specific rules of application in order to guarantee equitable access, quality of care, sustainable costs, professional liability, respect of patient privacy, data protection, and confidentiality. At present, telemedicine services could be only used as complementary or supplementary tools to the traditional healthcare services. Some indications for medical providers are suggested.

## 1. Introduction

In the 1970s, the term “telemedicine” was coined with the meaning of “healing at distance”, i.e., using Information and Communication Technologies (ICT) to improve patient outcomes by increasing access to care and medical information [1]. Later, a 1998 report of the World Health Organization (WHO) defined telemedicine/telehealth as: “Telemedicine is the delivery of health care services, where distance is a critical factor, by all health care professionals using information and communications technologies (ICT) for the exchange of valid information in diagnosis, treatment and prevention of disease and injuries, research and evaluation, and for the continuing education of health care providers, all in the interests of advancing the health of individuals and their communities, and that its purpose is to improve health outcomes” [2]. In addition, already at that time, the WHO pointed out that telemedicine presents special ethical problems such as maintaining the confidentiality of information and the privacy of patients, and safeguarding the integrity of information systems [2]. Indeed, this is what in fact occurred until the start of Coronavirus disease 2019 (COVID-19) pandemic emergency.

The most recent definition for telemedicine, telehealth, and related terms, which came out in 2020 from the US Centres for Medicare & Medicaid Services (CMS), is as follows: “the exchange of medical information from one site to another through electronic communication to improve a patient’s health” [3].

Although, according to the World Medical Association Statement on the Ethics of Telemedicine, ‘‘Face-to-face consultation between physician and patient remains the gold standard of clinical care’’ [4], in recent years, telemedicine has been increasingly practiced. Indeed, it provides several benefits, the most important of which include simplified access to health facilities and a reduction in the distance between patient and doctor, especially in geographical areas where the medical services are difficult to reach or in the case of seafarers, who are remote individuals [5]. Moreover, telemedicine may improve access to physicians for patients with mobility problems, such as patients with disabilities, fragile patients, or older patients [6], and could ideally promote equity of access to health care and quick patient engagement at reduced cost [1,7].

On the other side, teleservices such as teleanalysis, previously avoided as they were considered unsafe because of security concerns, are now recommended given the contemporary situation of many analysts in the world now being forced to work online due to the COVID-19 pandemic emergency [8,9].

Indeed, in this particular situation, telemedicine services have proven indispensable in facing the emerging needs of health care in this specific context [10]. Many lives have been saved during the COVID-19 pandemic through the use of telemedicine services making it possible to avoid physical or face-to-face contact with medical staff, healthcare personnel or other health professionals and patients in hospitals and clinical or health settings (unless strictly necessary), and by possibly reducing the virus spread and preventing or minimizing the risks of contagion either for patients or healthcare personnel [11,12].

The use of telemedicine may be also beneficial for the better management of medical care and diagnoses, and for the reduction of the duration of hospitalization in patients with no serious conditions [13,14] who can be treated in their homes, with higher-level virtually provided medical support or evaluation being available before hospital transfer, allowing the patients to possibly bypass the Emergency Department and be directly placed in a hospital bed [15].

Nevertheless, even in listing the recognized advantages of telemedicine or telehealth services, a series of ethical and legal issues may arise in the use of these disciplines and should be taken into consideration [7].

In the present review, aspects related to ethical or legal challenges dealing with telemedicine applied during the COVID-19 era are reported and presented in order to facilitate a better understanding of the related issues that still need a solution or a standardization across the countries, particularly with regards to patient privacy, informed consent, data protection, physicians’ liability and risk of malpractice, and laws and regulations.

## 2. Materials and Methods

A specific literature search was performed on PubMed using the following MeSH terms: Telemedicine (which includes Mobile Health or Health, Mobile, mHealth, Telehealth, eHealth), Ethics, Legislation/Jurisprudence, COVID-19. The terms were combined into a search string to retrieve more relevant literature with contents mostly focusing on the above-mentioned topics in the biomedical field (this was the reason behind the choice to use these MeSH terms). The search was set to retrieve articles with publication dates ranging from 1 March 2019 to 14 September 2021, and to identify only full texts in the English language.

Two authors independently screened the different types of articles by reading the abstracts (where available) and drafted a list of the studies that were likely eligible. Once all the full texts were retrieved, an additional selection was carried out, based on reading the complete text of the articles. As regards the articles not selected by both of the authors, a discussion followed, and a consensus was reached for the uncertainty related to some articles to be included in this review.

Besides the filters applied to the above-mentioned search string (i.e., publication date and languages), the other inclusion criteria were: (a) the type of study (e.g., literature reviews, research articles, commentaries); (b) studies not relevant to the aim of this review; (c) articles also considering population of remote patients to be treated with telemedicine.

The exclusion criteria also concerned factors such as: (a) other different types of literature (e.g., letters, editorials, perspectives, viewpoints, abstracts); (b) studies that took into consideration only technical and engineering aspects of medical devices used in telemedicine.

## 3. Results

Following the literature search, the total number of retrieved records was 85, 18 of which were review articles. A preliminary selection was carried out and 23 articles were excluded, with the potentially relevant articles at this stage being 62.

**Figure 1:**
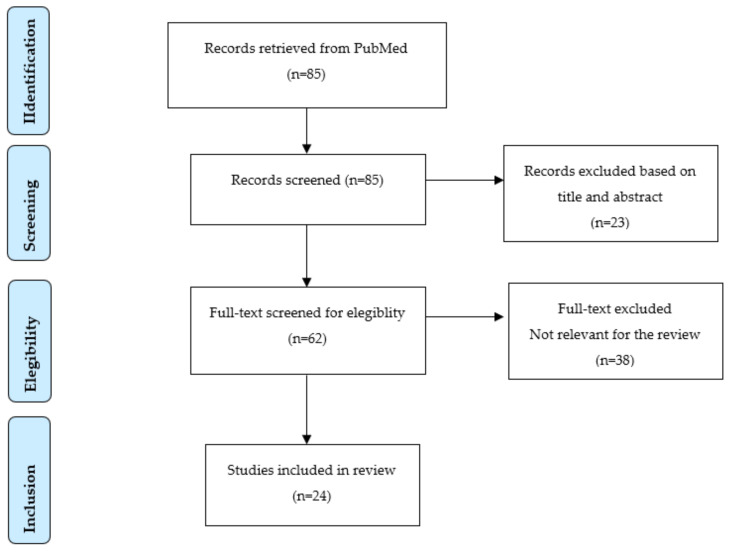
Study selection flowchart.

At the end of the full-text evaluation process, out of the previous 62 publications, 24 publications—including 14 reviews and 10 research articles—were considered eligible for inclusion (records excluded *n* = 38), based on their relevance to the aim of this review, namely “examining ethical and legal issues in Telemedicine practice during COVID-19 era” (Figure 1). The articles mainly considered telemedicine/telehealth services in general and across different countries.

The following medical fields, clinical routines, or non-urgent care types included in telemedicine/telehealth services emerged: Dermatology, Psychoanalysis and Psychotherapy, Pediatrics including perinatal and neonatal care, Nursing, Radiology, Neurology, Gynecology, Cardiology, Ophthalmology, Otorhinolaryngology, Orthopaedic and Musculoskeletal care, Nephrology, Endocrinology, Sports medicine, Chronic illnesses, COVID-19 care, and Follow-up care. This large list indicated that the application of telemedicine increased with respect to the number of medical fields during the COVID-19 pandemic, due to the risk of contagion and the consequent reduction in face-to-face contacts between patients and physicians.

Core themes related to ethical and legal issues in telemedicine/telehealth were identified and analyzed in the selected literature and questions still to be resolved were raised. The synthesis of the critical factors is reported in Table 1.

The concerns reported by the selected articles mostly pertain to the ethical and legal aspects (often interlinked), which were already highlighted in previous literature before the pandemic [7], but nevertheless remain crucial. These included the following: informed consent (information about the risks and benefits of remote therapy) and autonomy (87%), patient privacy (78%) and confidentiality (57%), data protection and security (74.%), malpractice and professional liability/integrity (70%), equity of access (30%), quality of care (30%), the professional–patient relationship (22%), and the principle of beneficence or being disposed to act for the benefit of others (13%) [1,6,8,10,11,16,17,18,19,20,21,22,23,24,25,26,27,28,29,30,31,32,33,34]. Non-maleficence is the principle of preventing harm by actively promoting confidentiality, safety/safeguarding, and security. Codes of conduct and codes of ethics should be put into practice [16,32,33].

**Table 1:** Studies about ethical and legal issues in telemedicine practice during the COVID-19 health emergency.

Medical or Health Service	Ethical and/or Legal Issues	Medical Purposes/Disciplines	Article Type	Location	Reference
Telemedicine and Telehealth	Data privacy, security and storage; Clear regulations/laws for legal security of patients and professionals; Informed consent: practice guidelines and standardized informed consent forms (risks and benefits of remote therapy and research) are recommended; Professional secrecy; Patients medical records availability; Noncompliance; Autonomy; Professional–patient relationship; Nonmaleficence and beneficence (minimize harm); Service quality and effectiveness; How frameworks, codes of conduct, or guidelines, are being used to improve ethical telehealth practice; Confidentiality; Liability; Reimbursement; Access for rural/remote populations, patients of diverse races, ethnicities, socioeconomic statuses (equity, justice); Licensing requirements; Risk of malpractice and insurance coverage; Establishment of protocols for managing laboratory tests, prescriptions, and scheduling; Recording issues: doctors must obtain consent before recording, patients do not need a doctor’s consent to record a consultation; Tailoring services to each patient; Patient and clinicians responsibilities; Commercialization; Cybersecurity and software safety; Evaluation as an ethical imperative (sound evidence on which to base analyses, decisions, and services); Compliance with ethical principles; Legality of patient recording depends on the state or territory: in certain jurisdictions, patients can secretly record a consultation without the consent of the clinician and this recording may be used in legal or disciplinary proceedings; Patient’s medical records will generally be held and owned by the clinician or health care organization, but patients are entitled to access and take a copy of their records; Safeguarding risks (patient self-harm); Safety (such as how to deal with a patient falling in their home during a consultation): guidance to ensure the safety of patients and clinicians in delivering virtual consultations is needed; Health Insurance Portability and Accountability Act (HIPAA) law revision; Fidelity and responsibility (trusting relationships); Integrity (no fraudulent behavior nor personal gain); Respect for people’s rights and dignity (protect privacy and safeguarding); Need of ethics code; Boundaries of competence; Unfair discrimination in treatment delivery; Digital communication with patients must be compliant with the country’s and organization’s data protection and telehealth regulations that are rapidly evolving and subject to change.	Nursing; Radiology; Neurology; Dermatology; Psychotherapy and mental health. Clinical and routinehealth care; Follow-up care and of chronic diseases; Chronic medical illnesses and malignancies care; Visits to determine the urgency of medical or surgical interventions; COVID-19 severe cases screening; Monitoring clinically stable patients; Maintaining outpatient care; Mental health care; Radiology; Cardiological diagnosis; Dermatology care; Ophthalmology care; Otorhinolaryngology care; Non urgent clinical care; Second opinions and medical check-ups; Complementary service to face-to-face consultations; Virtual Orthopaedic and Musculoskeletal care; Daily clinical practice such as perinatal and neonatal care; Preventive care; Diagnosis and treatment of a health condition; Psychological care; Management of diabetes, hypertension, asthma, stroke, cancers, and chronic pain; Triage and management of a wide range of acute conditions.	Systematic Review; 5 Reviews; Special Article; Ethics and Law article; Research Article; Legislation Article; Legal issues and risk management article; Ethics Article; Recommendations	Various, USA; India; Brazil; UK; Various; Spain; Australia;	[16] [19] [21] [23] [26] [11] [6] [28] [29] [30] [31] [33] [34]
Pediatric and adolescent telehealth; Behavioral telehealth.	Privacy and security challenges; Inequitable access to care; Unsustainable costs in a fee-for-service system; Lack of quality metrics for novel care-delivery modalities; Telehealth policies and regulations (e.g., payer restrictions on telehealth reimbursement, complex medication-prescribing regulations for virtual care).	Pediatric ambulatory care; Adolescent care; Screening; Presurgical visit; Chronic condition management; Follow-up appointments; Behavioral and mental health (ADHD, Depression).	Review	USA	[10]
Telemedicine for abortion	Informed consent; Safeguarding support; Good-quality care; Equity of access.	Abortion medication and care.	Review (viewpoint)	Various	[17]
Teledermatology	Informed consent; Medical ethics; Lack of personal relationship between the patient and the dermatologist is a main ethical concern; Strictness of law varies from country to country; Malpractice or risk of telemalpractice (phantom patient); Risk of abuse and breaches of patient confidentiality; Patient’s autonomy; Privacy; Data protection.	Dermatology care and treatment; Chronic skin conditions with co-morbidities; Skin disorders; Skin diseases care; Diagnostic and treatment purposes; Follow-up appointments.	2 Reviews; Research Article.	India; Various.	[18] [20] [27]
Teleneurology	Malpractice coverage; Data protection; Informed consent; Patient privacy; Validation and development of best practice standards.	Neurology outpatient care; Follow-up visits.	Review	USA	[22]
Sport and exercise medicine telehealth	Ensure patient safety; Secure and effective communication methods; Ensure that patient feedback mechanisms are in place; Evaluate and ensure patient satisfaction; Informed care and shared treatment decisions; Promoting open communication and consent; Mutual respect; Access to health information; Physician autonomy and responsibilities.	Clinical care in the broad field of sports medicine; Follow-up consultations.	Review	Various	[24]
Telepsychiatry, Teleanalysis or Teletherapy	Telemedicine use; Privacy; Confidentiality; Data protection; Security; Informed consent; Physician’s malpractice and liability. Patient benefice; Justice (support/access is variable); Autonomy; Licensing and reimbursement; New guidelines to ensure patient privacy and quality of care.	Psychiatric and mental health care services and assistance; Psychoanalysis; Psychotherapy.	Review; Healthcare ethics article	Various; USA.	[25] [32] [8]
Telecardiology; Telemonitoring in dialysis; Telemonitoring in diabetes; Perinatal Telemonitoring.	Authorization and accreditation; Protection of patient confidentiality; Professional liability (e.g.,: incorrect diagnosis: erroneous reading of the report or to the poor quality of transmitted images); Absence of specific regulatory provisions; Physician–patient relationship; Privacy; Informed consent; Data sharing; Malpractice; Information security; Patient self-determination; Standardization of the practices; Economic reimbursement.	COVID-19 care; Chronically ill out-of-hospital patients (cardiology, diabetes); Nephrology; Endocrinology; Gynecology.	Commentary	Italy	[1]

Law/regulations/legal issues (83%) stress the absence or variation of the rules among countries and the need for guidelines/best practices or standardization of telemedicine services. In particular, the questions raised regarded the following aspects: costs of services and reimbursement, insurance coverage, virtual prescription of medications, accreditation, licensing, commercialization, recording (as an area of controversy), and evaluation of the effectiveness of the services such as health outcomes and delivery, in terms of quality and cost, individual experience, program implementation, and key performance indicators [1,6,8,10,11,16,19,21,22,23,24,27,28,29,30,31,32,34].

Remote patients could either benefit or be disadvantaged by virtual care (e.g., lack of access to Internet, smartphones, or other technology should not prevent children from accessing their medical system) [10]. Indeed, the principle of justice includes equal access to care and fair distribution of the technology for marginalized communities [16]. Ideally, the greatest advantage for patients should be the equitable and quick access to healthcare through telemedicine services, but this aspect is still controversial, and in some cases, has been exacerbated during the COVID-19 pandemic (e.g., inequitable access to care, unsustainable costs in a fee-for-service system, and a lack of quality metrics for novel care-delivery modalities). Therefore, the practice of telemedicine needs a strong improvement, with specific rules and codes of conduct to be correctly put in practice for a sustainable program to be built.

## 4. Discussion

The selected literature highlighted important issues that have to be considered in the application of telemedicine or telehealth services. Among these, informed consent in telemedicine must have the same basic requirements as for traditional medical services. Therefore, there should be a substantial equivalence between telehealth and traditional services. Indeed, telemedicine should correspond to a different way of providing health and social health services but with similar results as the traditional face-to-face approaches.

The implications should be discussed in the broadest context possible. Moreover, the evolution of telemedicine poses a series of legal or ethical problems ranging from authorization and accreditation profiles to the protection of patient’s personal data and many other critical aspects that need require standardization and regulatory processes [7,35]. In this regard, the Italian National Institute of Health (Istituto Superiore di Sanità) recently published a report on COVID-19. Indications on telemedicine healthcare services during the COVID-19 health emergency were provided and recommended the proactive monitoring of the health conditions of people in quarantine, those in isolation or who have been discharged from the hospital, or those isolated at home, who are limited by the rules of social distancing but still in need of continuity of care, even if they are not COVID-19-infected [36]. Moreover, a practice pointer was proposed, summarizing the evidence on the use of video consultations in primary and specialist care during the COVID-19 pandemic and offering practical recommendations for video consulting in outpatient settings [34].

However, at present, the characterization of the ethical and legal issues and their solution, in the absence of a specific set of regulations, is mainly left to the assessment of compatibility between the practices adopted so far and the general regulatory framework [1].

The evidence of the effectiveness of video consultations is poor, but points towards effectiveness, safety, and high satisfaction in patients and healthcare providers [34]. The evaluation of telemedicine services is a priority of future research and a fundamental action to ensure the quality of the service and, as a consequence, the final adoption and implementation of the service at full capacity. Until then, telehealth should be a supplementary method and not a substitute of face-to-face methods of health care delivery [16].

With respect to the legal issues of virtual prescription of medication, recently highlighted by Curfman et al. [10], it is worth noting that telepharmacy (“a form of pharmaceutical care in which pharmacists and patients are not in the same place and can interact using information and communication technology (ICT) facilities”) also presents unresolved limitations (e.g., legal implications) [37]. In Italy, a recent investigation stressed the need for more efforts to be made by national public health stakeholders to better analyze the contribution of telemedicine services available in public pharmacies and to find the best solutions to implement this innovative technology as an established service [38].

However, despite the difficulties, in the U.S., there are websites that are providing information for health care providers and patients who are geographically isolated, or economically or medically vulnerable, and promote virtual health care (telehealth), such as the one built by the Health Resources and Services Administration (HRSA), which provides legal considerations and best practice guides [39]. The American Medical Association (AMA) is also providing a Code of Medical Ethics in Telemedicine Practice [40], and several other organizations such as the American Psychological Association on are providing information on how to conduct group therapy using telehealth during COVID-19 [41]; in addition, the Kaiser Family Foundation (KFF), a nonprofit organization that provides independent information on national health issues, is providing information about opportunities and barriers for telemedicine in the U.S. during the COVID-19 emergency [42]. Telehealth could be practiced by video visits, phone calls, online communication such as email or text messaging, with careful attention paid to the secure storage of patient data (images, lab results, or vital statistics).

Some of the indications for medical providers include:

- Conducting telehealth in private settings, such as a doctor in a clinic or office connecting to a patient who is at home or at another clinic. Providers should always use private locations and patients should not receive telehealth services in public or semi-public settings, absent patient consent or exigent circumstances;
- Obtaining patients’ consent verbally and noting it in the medical record. For a signed form, the patient portal or the mail should be used to obtain a signature. It is not necessary to wait for a signed consent form. A telehealth visit can be conducted with patients giving their consent verbally.
- Treat telehealth appointments in the same way as an in-person appointment and the patient should not hesitate to ask questions and request explanations or clarifications [39].

Finally, it has to be said that the review presented here has some limitations. The main limitation is that it is a narrative review conducted using only one electronic database (PubMed) to search for articles using MeSH terms. This decision was made in order to make it possible to rapidly review the literature related to the contribution of telemedicine service during the current pandemic situation, and to give an overview of the legal and ethical issues raised in the practice of the service. Although this possibly limited the number of references obtained, we are confident that the reported information covers the issues that need to be faced quite thoroughly, and highlights the fact that telemedicine is considered an indispensable service to be used.

## 5. Conclusions

Currently, according to the literature herein reviewed, the ethical and legal issues related to the practice of telehealth or telemedicine services still need standard and specific rules of application in order to guarantee equitable access, quality of care, sustainable costs, professional liability and respect of patient privacy, and data protection and confidentiality. In fact, telemedicine services could be used only as complementary or supplementary to the traditional healthcare services and not as a complete substitute.

Nevertheless, telemedicine has the potential to have widespread applications and health professionals play a fundamental role in terms of following rigorous indications when conducting telehealth visits and in helping to ensure that these technologies respect the therapeutic relationship and the quality of care.

## Data Availability

Not applicable.
